# Online Behavioral Addictions

**DOI:** 10.1192/j.eurpsy.2025.35

**Published:** 2025-08-26

**Authors:** M. N. Potenza

**Affiliations:** 1 Psychiatry; 2 Child Study Center; 3 Neuroscience, Yale School of Medicine, New Haven, United States

## Abstract

**Abstract:**

Disorders due to addictive behaviors have been included in the eleventh revision of the International Classification of Diseases (ICD-11). Such conditions include gambling disorder and gaming disorder, and each has online specifiers given the relevance of the internet to each of these conditions in modern societies. This presentation will consider problematic usage of the internet with respect to formally specified disorders (gambling, gaming) as well as other online behaviors (e.g., use of social media) for which problematic engagement may be considered as an “other specified disorder due to addictive behaviors” in the ICD-11. European and global initiatives (e.g., the Lancet Psychiatry Commission on Problematic Usage of the Internet and the development of screening and diagnostic instruments involving World Health Organization workgroups) will be discussed. Clinical, developmental and public health implications will be presented to provide an up-to-date understanding of rapid changes in this area.

**Disclosure of Interest:**

None Declared

